# Enhancing deep learning classification performance of tongue lesions in imbalanced data: mosaic-based soft labeling with curriculum learning

**DOI:** 10.1186/s12903-024-03898-3

**Published:** 2024-02-01

**Authors:** Sung-Jae Lee, Hyun Jun Oh, Young-Don Son, Jong-Hoon Kim, Ik-Jae Kwon, Bongju Kim, Jong-Ho Lee, Hang-Keun Kim

**Affiliations:** 1 Department of Biomedical Engineering, College of IT Convergence, Gachon University, Seongnam, Republic of Korea; 2 Oral Oncology Clinic, National Cancer Center, Goyang, Republic of Korea; 3 Neuroscience Research Institute, Gachon Advanced Institute for Health Science and Technology, Gachon University, Incheon, Republic of Korea; 4 Department of Psychiatry, Gachon University College of Medicine, Gil Medical Center, Incheon, Republic of Korea; 5 Department of Oral and Maxillofacial Surgery, Seoul National University Dental Hospital, Seoul, Republic of Korea; 6 Dental Life Science Research Institute, Seoul National University Dental Hospital, Seoul, Republic of Korea; 7 Dental Research Institute, Seoul National University, Seoul, Republic of Korea

**Keywords:** Tongue cancer, Class imbalance, Deep learning, Mosaic augmentation, Curriculum learning

## Abstract

**Background:**

Oral potentially malignant disorders (OPMDs) are associated with an increased risk of cancer of the oral cavity including the tongue. The early detection of oral cavity cancers and OPMDs is critical for reducing cancer-specific morbidity and mortality. Recently, there have been studies to apply the rapidly advancing technology of deep learning for diagnosing oral cavity cancer and OPMDs. However, several challenging issues such as class imbalance must be resolved to effectively train a deep learning model for medical imaging classification tasks. The aim of this study is to evaluate a new technique of artificial intelligence to improve the classification performance in an imbalanced tongue lesion dataset.

**Methods:**

A total of 1,810 tongue images were used for the classification. The class-imbalanced dataset consisted of 372 instances of cancer, 141 instances of OPMDs, and 1,297 instances of noncancerous lesions. The EfficientNet model was used as the feature extraction model for classification. Mosaic data augmentation, soft labeling, and curriculum learning (CL) were employed to improve the classification performance of the convolutional neural network.

**Results:**

Utilizing a mosaic-augmented dataset in conjunction with CL, the final model achieved an accuracy rate of 0.9444, surpassing conventional oversampling and weight balancing methods. The relative precision improvement rate for the minority class OPMD was 21.2%, while the relative ${F}_{1}$ score improvement rate of OPMD was 4.9%.

**Conclusions:**

The present study demonstrates that the integration of mosaic-based soft labeling and curriculum learning improves the classification performance of tongue lesions compared to previous methods, establishing a foundation for future research on effectively learning from imbalanced data.

## Introduction

Oral cavity cancer accounted for approximately 377,000 new cases and 177,000 related deaths worldwide in 2020 [1], highlighting its significance as a public health issue. Tongue cancer is frequently diagnosed in many countries, making it an important area of focus. While oral cavity cancer is associated with high morbidity and mortality rates [2], oral potentially malignant disorders (OPMDs) can also increase the risk of developing this type of cancer, including tongue cancer [3, 4]. Therefore, it is essential to accurately and easily diagnose oral cavity cancers and OPMDs to prevent their progression. Accurate and accessible diagnosis techniques can lead to timely treatment and reduce cancer-specific morbidity and mortality [5–7].

Recently, the development of artificial intelligence has led to the use of deep learning to detect oral cavity cancers and OPMDs [8–15]. VGG [16], ResNet [17], and EfficientNet [18] techniques were commonly utilized in these studies. Most studies [11–14] have focused on binary classification, classifying oral lesions as either malignant or benign. Only a few studies [15] have investigated multi-class classification. Recent work showed that it is recommended to use a moderately complex convolutional neural network (CNN) with a data-bypassing architecture when working with a limited dataset. Nevertheless, one of the remaining challenges is addressing the class imbalance [19, 20]. Class imbalance, a prevalent issue in medical imaging applications, especially for cancer detection, occurs when certain classes are disproportionately represented in a dataset. This disparity can degrade classifier performance by neglecting minority classes [21–24]. In this study, the dataset was imbalanced, with the cancer and OPMD categories having fewer samples.

While the representativeness of a dataset is crucial for the effectiveness of deep learning algorithms, class imbalance is a frequent challenge in medical imaging applications, making it difficult to acquire representative datasets. Consequently, it is imperative to develop methods that enable the effective training of deep learning models on imbalanced and limited datasets. The goal is to ensure these models can achieve performance levels comparable to those trained on representative datasets. Several approaches have been proposed to address this issue, including data-level, algorithm-level, and hybrid methods [20, 25]. An effective data-level method is data augmentation, which can increase the diversity of a training dataset by applying data transformations. Examples of these augmentation techniques include Cutout [26], CutMix [27], Random Image Cropping and Patching (RICAP) [28], and Mosaic augmentation [29]. Cutout and CutMix techniques can enhance the performance of machine learning models by manipulating important parts of the input data. This helps the model to learn more robust features, resulting in better performance. These techniques have shown promise when applied to various models and datasets, making them a promising area for future machine learning research. RICAP is an augmentation technique that enhances the diversity of training datasets. It uses four random crops from the input images and patches them together to form a single image. This approach enables the model to learn from more diverse data and helps prevent overfitting. However, a significant limitation of RICAP, particularly in tongue cancer detection, is its propensity to lose lesions in the produced images. This problem occurs mainly due to the small size of the lesions and their frequent placement on the lateral edges of the tongue, which may be accidentally excluded during the random cropping process. Mosaic augmentation combines four image patches and resizes them into a single synthesized image to detect objects that may not be easily recognizable in their normal context owing to differences in scale [30]. Although Mosaic augmentation has proven to be effective in detecting objects, it is important to note that it was originally designed for object detection and may not be directly applicable to classification tasks.

In this study, we introduced an effective technique called "mosaic-based soft labeling" augmentation combined with curriculum learning (CL) [31]. CL begins with teaching the model using simpler training dataset (or task) and then gradually introduces more complex training dataset (or task). This method mimics human learning, allowing the model to build on previously learned concepts from simpler dataset, thereby facilitating the understanding of more complex concepts more accurately. Using this approach, augmented data with different levels of complexity are trained step-by-step accordingly. The purpose of this study was to evaluate the performance of mosaic-based soft labeling and CL compared to other methods, such as oversampling and weight balancing, in the class-imbalanced tongue lesion dataset.

## Methods

### Dataset

From January 2006 to December 2020, a total of 1,810 tongue images were acquired from patients aged over 20 years who visited the Department of Oral and Maxillofacial Surgery at Seoul National University Dental Hospital in Seoul, Republic of Korea, for diagnostic purposes or periodic check-ups. The clinical photographs were consistently captured using a single-lens reflex camera (D750, Nikon, Japan) by a single researcher under the supervision of the author (JH Lee). The captured images were saved in JPEG (Joint Photographic Experts Group) format. The Institutional Review Board of the Seoul National University Dental Hospital approved the collection and use of this dataset (ERI22034).

The images were categorized into three distinct groups with an average resolution of 723 × 734 pixels: cancer, OPMD, and noncancerous lesions (Table 1). The images were categorized into three distinct groups with an average resolution of 723 × 734 pixels: cancer, OPMD, and noncancerous lesions (Table 1). Notably, the dataset used in this study consists of cases from the cancer class (20.6%), OPMD class (7.8%), and noncancerous lesion class (71.6%), with a significant imbalance between classes. Tongue cancer was defined as squamous cell carcinoma confirmed through pathological examination. OPMDs were diagnosed in accordance with the World Health Organization consensus report classification [3]. Lesions including pathologically confirmed oral lichen planus, leukoplakia, or erythroplakia were classified as OPMDs. Noncancerous lesions encompassed healthy tongue tissues and benign conditions such as hemangioma, fibroma, or aphthous ulcer. Two licensed surgeons with a minimum of 5 years of clinical experience (IJK and JHL) were responsible for the categorization of tongue images. For the test set, 10% (180 images) of the entire dataset was randomly selected to reflect the data population. The remaining dataset was used as training and validation datasets at 70% and 30%, respectively.

**Table 1:** Categories and number of tongue lesion samples

Categories	Number of samples
cancer	372
OPMD	141
noncancerous lesion	1,297
Total	1,810

### Data augmentation: mosaic-based soft labeling

**Fig. 1:**
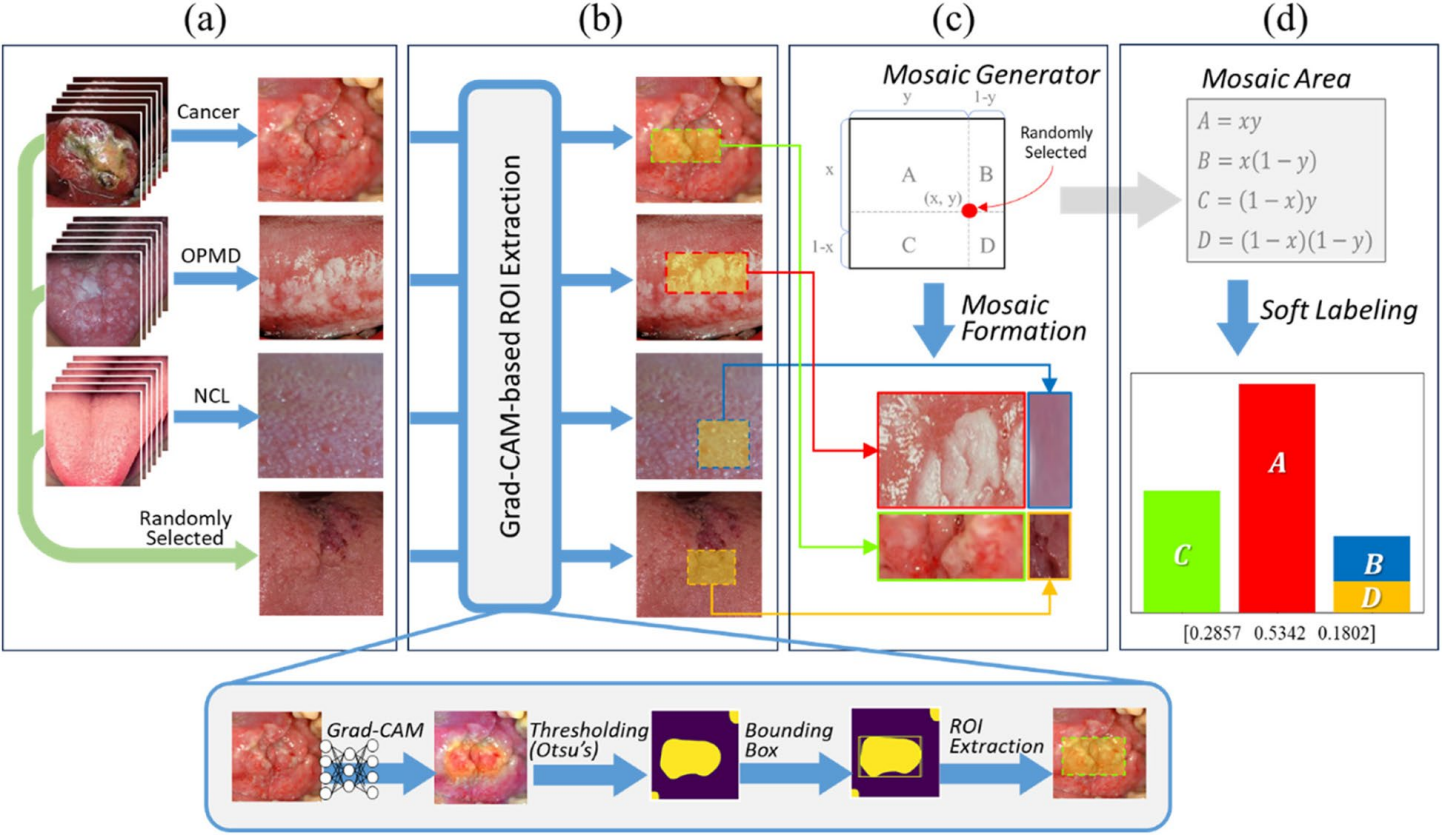
A detailed description of mosaic formation and soft labeling process. **a** A set of four images consisting of a mosaic image. One image is selected for each class and the last one is additionally and randomly selected from all classes (in this case, the noncancerous lesion). **b** A Grad-CAM-based ROI extraction proposes a representative region of each image. A class activation map of each image is extracted from the trained model without mosaic dataset. **c** The mosaic generator randomly chooses a point within a square to form a 2 × 2 grid so that one segment should be, at least, larger than half of the synthesized image. A mosaic image is synthesized with four cropped and rescaled images from each Grad-CAM-based ROI. The position of each image is randomly assigned each time a synthesized image is created. Also, the oversampling rate is adjustable, allowing for larger areas to be allocated to user-defined classes. (in this case, A: OPMD, B: noncancerous lesion, C: cancer, D: noncancerous lesion) **d** The soft label is calculated with the areas of each class within the grid. The model is trained to accurately predict the value of each component in the soft label vector

To address the class imbalance and limited quantity of training data, we proposed a new augmentation technique named “mosaic-based soft labeling” (Fig. 1). This technique was motivated by two existing methods: *RICAP* [28] and *mosaic data augmentation* [29]*.* However, in contrast to these approaches, we used Gradient-weighted Class Activation Mapping (Grad-CAM) [32] to extract the representative regions of each image. Grad-CAM calculates the relationship between output and input images by using a gradient. This produces a heatmap that emphasizes the areas of the input image used by the network to determine the target class. Otsu’s thresholding converts the Grad-CAM importance map into a binary map consisting of representative and nonrepresentative regions. Next, we determined the bounding box that covers the largest representative region in the resulting binary map. Finally, we combined the representative patches using cropping and resizing, which helped train the network to recognize representative patterns in the imbalanced data. The resulting synthesized image forms a mosaic-based soft labeling dataset.

In Mosaic augmentation, four images are combined to create a single synthesized image. However, because our goal was to classify the data into three categories, we selected one patch from each class and randomly chose the remaining patches using random sampling with replacement. The four selected representative patches were then combined to form a synthesized image. The label was calculated using the area covered by each class, with a sum of 1. The portion of the area occupied by each class can be interpreted as a probability associated with that specific class, intrinsically utilizing the soft labeling technique [33]. The model is trained to accurately predict the value of each component in the soft label vector. The soft labeling technique replaces the one-hot ground truth with smoothed labeling, which is known to improve classification performance by decreasing overconfidence and increasing generalization.

### Training: CL using the mosaic-based soft labeling

**Fig. 2:**
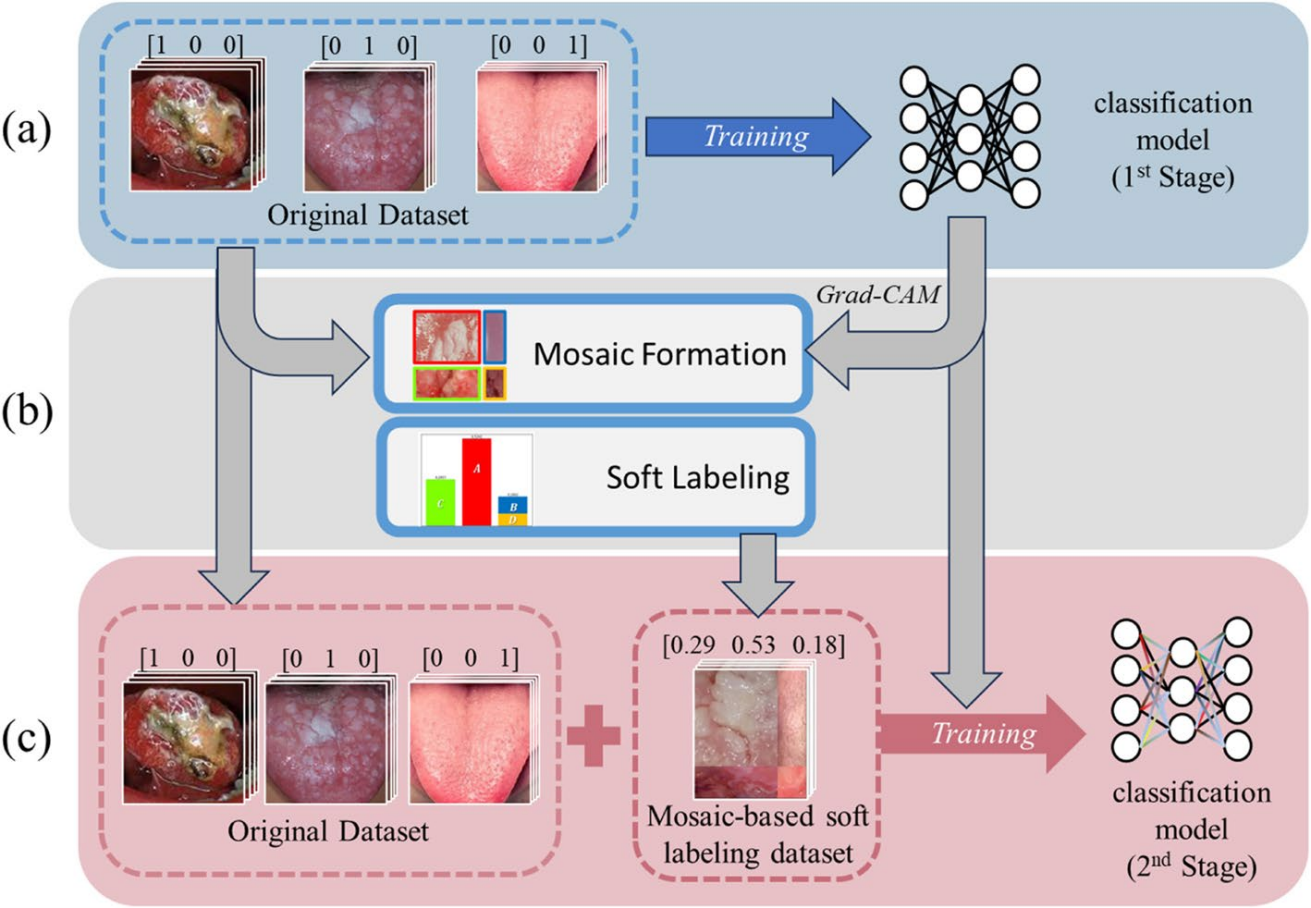
An overview of mosaic-based soft labeling with curriculum learning. Our curriculum learning is consisted of two stages. **a** In stage 1, a conventional training is performed using an original dataset with the weight balancing method. Transfer learning, data augmentation, and fine-tuning was also used during training. **b** Using the trained model in Stage 1, a mosaic dataset and the corresponding soft labels can be obtained as described in Fig. 1. **c** In stage 2, the final model is trained with the original dataset and a newly added mosaic-based soft labeling dataset

CL consists of several different training stages, each of which becomes progressively more challenging. In our study, we used CL consisting of two stages. Stage 1 is a conventional training using an original dataset with the weight balancing method. 1,630 images from the dataset are utilized, excluding the 180 images reserved for the test set. Mosaic-based soft labeling dataset is created using Grad-CAM with the model trained in Stage 1. In Stage 2, the model is retrained using a newly added mosaic-based soft labeling dataset and the original dataset. 1,200 mosaic-based soft labeling images (synthesized images) are utilized. Note that the test set of 180 images is not used for training of either stage. An overview of our CL is shown in Fig. 2.

The details of our CL stage 1 are as follows. The EfficientNet model was used as a feature extraction model for classification. This model outperforms other existing CNNs with similar computational costs by utilizing a scaling method that uniformly scales up the width, depth, or resolution [18]. The EfficientNetB0 model was used in this study. Deep learning models must be trained from scratch when the training data have different feature spaces and data distributions, which leads to an inefficient situation. When the available training dataset is relatively small, transfer learning is an effective method for training deep models without overfitting, and it has been used in many studies [34–37]. Transfer learning involves reusing learned knowledge from a base dataset; typically, a large-scale dataset such as ImageNet is used [38]. In this study, transfer learning was performed using pretrained weights from ImageNet [38]. Conventional data augmentation was applied to the training dataset to increase its diversity. The Inputs were zoomed in by 80%-100% at random and flipped horizontally and vertically. They were rotated at random angles ranging from –30° to 30°. In addition, they were shifted to the left or right at random between -10% and 10% of the total width, and vertically in the same manner. Outside the input boundaries, the points were filled with the nearest pixels. Finally, fine-tuning was performed to train the network on the tongue cancer dataset. After fine-tuning, the network was retrained entirely on the tongue cancer dataset with a low learning rate owing to the significant differences between the ImageNet and tongue cancer datasets. This approach is commonly used when applying deep neural networks to medical applications and helps overcome the scarcity of medical datasets [35, 39]. Furthermore, because of the highly imbalanced class distribution of the datasets, a weight balancing method [40] was utilized. This method heavily penalizes misclassified predictions from the minority class and is adopted to address the class imbalance issue. At the end of Stage 1, the mosaic-based soft labeling dataset is created in the manner of the Mosaic-based soft labeling section.

In Stage 2 of our CL, the model resulting from Stage 1 was retrained using an augmented dataset that included both the original tongue cancer dataset and the mosaic-based soft labeling dataset. The weights are initialized and trained from the beginning. For the mosaic-based soft labeling dataset, the rotation and flipping were applied to eliminate the effect of cross-shaped lines between images in a synthesized image. The rotation range was set at random angles between -5° and 5°.

### Test: prediction of the trained model

In the field of machine learning, including deep learning, prediction refers to the process of using a trained model to make a classification or regression on new data that was not included in the training set [41]. Prediction is also called inference. In this study, we have trained three deep learning models, each sharing the same underlying architecture but differing in the applied training technique. The first model employs a weight balancing technique, the second utilizes oversampling technique, and the third is trained using our proposed method, which integrates mosaic-based soft labeling and CL.

To assess performance, all three models are tested using a uniform test set, with comparative metrics derived from the outcomes of this test, which fundamentally involves an inference process. As described in the Dataset section, the test set comprises images that are not utilized during the training. It is noted that the mosaic images generated through mosaic-based soft labeling are not included in the test set.

**Fig. 3:**
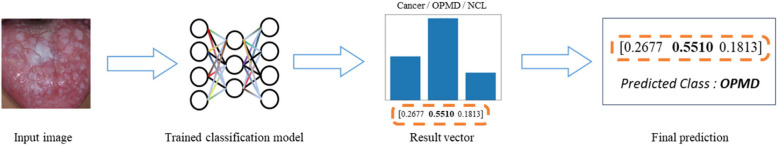
Prediction process of the proposed method. During the prediction stage, where the models are fed only real images, the model produces a result vector for each input image. The image is classified into the class corresponding to the highest value component in its result vector

Figure 3 shows how the prediction works in our proposed method. During the prediction stage, where the models are fed only real images, each model produces a result vector for each input image. Subsequently, each image is classified into the class corresponding to the highest value component in its result vector. In contrast, the training stage uses both real and synthetic images. The result vector itself is considered the final output of each model. This vector is then compared with the corresponding label — a soft label for synthetic images and a one-hot encoding label for real images — to calculate the loss function, which are then minimized through continued training.

### Metrics and statistical analysis

We computed the accuracy, precision, recall, and ${F}_{1}$ score using a test set to assess model performance. Accuracy is the ratio that indicates how accurate the classification was among all classification attempts. Accuracy ranges from 0 to 1. A score of 0 suggests no accuracy, meaning all classifications are incorrect, while a score of 1 indicates perfect accuracy with all classifications being correct. Accuracy is a commonly used metric for evaluating classification models because of its simplicity. However, it may not be suitable for imbalanced datasets [42]. Therefore, additional metrics to complement basic accuracy need to be considered. Precision is a measure that indicates the accuracy of positive predictions, specifically for a particular class. It shows how many of the predicted instances for that class are actually true instances of that class. On the other hand, recall, also known as sensitivity, is a measure that indicates the ability to detect all instances of a specific class through predictions. It shows how many of the total instances of that specific class are correctly identified. ${F}_{1}$ score is a single metric that combines both precision and recall into a single value, providing a balanced measure of a model's performance in classification tasks, especially when there is an imbalance between the classes. It is particularly useful when you want to find a balance between precision and recall, as they are often in tension with each other. Precision, recall, and ${F}_{1}$ score range from 0 to 1, with 0 indicating the worst possible performance and 1 indicating the best possible performance.

In this paper, we performed multi-class classification with three categories. Therefore, we calculated weighted average and macro average (Eq. 3) for precision, recall, and ${F}_{1}$ score, based on the averages of each class.

$$\mathrm{macro}\;\mathrm{average}=\frac13\times({\text{score}}_{\mathrm{cancer}}+{\text{score}}_\text{OPMD}+{\text{score}}_{\mathrm{noncancerous}\;\mathrm{lesion}})$$

$$\mathrm{weighted}\;\mathrm{average}={{\text{W}}_\text{cancer}\times\text{score}}_{\mathrm{cancer}}+{\text{W}}_\text{OPMD}\times{\text{score}}_\text{OPMD}+{{\text{W}}_{\mathrm{noncancerou}s\;\mathrm{lesion}}\times\text{score}}_{\mathrm{noncancerous}\;\mathrm{lesion}}$$

where

$$\begin{array}{l}{\text{W}}_\text{cancer}=\mathrm{number}\;\mathrm{of}\;\mathrm{cancer}\;\mathrm{samples}\;\mathrm{divided}\;\mathrm{by}\;\mathrm{number}\;\mathrm{of}\;\mathrm{total}\;\mathrm{samples}\\{\text{W}}_\text{OPMD}=\mathrm{number}\;\mathrm{of}\;\mathrm{OPMD}\;\mathrm{samples}\;\mathrm{divided}\;\mathrm{by}\;\mathrm{number}\;\mathrm{of}\;\mathrm{total}\;\mathrm{samples}\\{\text{W}}_{\mathrm{noncancerous}\;\mathrm{lesion}}=\mathrm{number}\;\mathrm{of}\;\mathrm{noncancerous}\;\mathrm{lesion}\;\mathrm{samples}\;\mathrm{divided}\;\mathrm{by}\;\mathrm{number}\;\mathrm{of}\;\mathrm{total}\;\mathrm{sample}\end{array}$$

Weighted average is a metric calculation that considers the contribution of each class or category in proportion to its prevalence in the dataset. It assigns more weight to classes with larger sample sizes, which is beneficial when dealing with imbalanced datasets. Weighted averages are commonly used in classification tasks. However, in cases of class-imbalanced data, it may not be appropriate. In contrast, the macro average is a metric calculation that independently computes the metric for each class and then calculates the unweighted average (simple arithmetic mean) of those class-specific metrics. It treats all classes equally, regardless of their prevalence in the dataset, providing a balanced assessment of model performance across all classes. Macro average is often used to evaluate the overall model performance when all classes are considered equally important.

We also used the relative performance improvement rate as an indicator of how much the model’s performance improved compared to other conventional methods (Eq. 4).

$$\mathrm{relative}\;\mathrm{performance}\;\mathrm{improvement}\;\mathrm{rate}(\%)=100\times\frac{({\text{Performance}}_\text{new}-{\text{Performance}}_\text{previous})}{{\text{Performance}}_\text{previous}}$$

## Results

Table 2 shows the accuracy comparison across three different models, while Table 3 presents their other performance metrics.

**Table 2:** Accuracy comparison among weight balancing technique (WB), oversampling technique (OS), and mosaic-based soft labeling (MBS)

Accuracy		
**WB**	**OS**	**MBSO**
0.9278	0.9111	**0.9444**

**Table 3:** Classification performance with weight balancing technique (WB), oversampling technique (OS), and mosaic-based soft labeling (MBS)

	**precision**			**recall**			${{\varvec{F}}}_{1}$ **score**		
	**WB**	**OS**	**MBS**	**WB**	**OS**	**MBS**	**WB**	**OS**	**MBS**
cancer	0.8293	0.8250	**0.8500**	**0.9189**	0.8919	**0.9189**	0.8718	0.8571	**0.8831**
OPMD	0.6875	0.6364	**0.8333**	**0.7857**	0.5000	0.7143	0.7333	0.5600	**0.7692**
noncancerous lesion	**0.9919**	0.9612	0.9844	0.9457	0.9612	**0.9767**	0.9683	0.9612	**0.9805**
weighted average	0.9348	0.9080	**0.9450**	0.9278	0.9111	**0.9444**	0.9302	0.9086	**0.9441**
macro average	0.8362	0.8075	**0.8892**	**0.8835**	0.7844	0.8700	0.8578	0.7928	**0.8776**

The first model (WB), which was only trained up to Stage 1, used the conventional weight balancing technique to address the class imbalance. The overall accuracy of Stage 1 was 0.9278. The precision and ${F}_{1}$ score of OPMD (minority class) were 0.6875 and 0.7333, respectively.

Oversampling (OB) is another well-known technique to address class imbalance. The second model was trained using the oversampling method instead of the weight balancing method, while following the same procedure as the first model. Cancer and OPMD images were sampled using replacement to match the number of the majority class. The overall accuracy of oversampling was 0.9111. The oversampling method showed poorer performance on each metric when compared to the weight balancing method.

The final model (MBO), which was trained using the newly added mosaic-based soft labeling dataset (Stage 2), achieved an accuracy rate of 0.9444. This outperformed conventional oversampling (0.9111) and weight balancing methods (0.9278). Our approach demonstrated comparable or slightly lower performance in various categories compared to conventional methods. However, it significantly improved performance in the OPMD category, which is characterized by a notably small sample size. We saw a 21.2% increase in relative precision improvement and a 4.9% increase in relative ${F}_{1}$ score improvement rate when compared to conventional weight balancing method (precision: 0.6875 → 0.8333; ${F}_{1}$: 0.7333 → 0.7692). This suggests that our method trains the classifier model more effectively when handling class-imbalanced training data, compared to traditional approaches.

## Discussion

Medical data are frequently imbalanced, which can negatively affect classification performance because the model may not properly capture the minority class [43]. To address this issue, various methods have been introduced, including oversampling and weight balancing. Our proposed method, when tested on an imbalanced tongue lesion dataset, yielded an accuracy rate of 0.9444. This represents a modest improvement over the accuracy rates of conventional oversampling (0.9111) and weight balancing methods (0.9278). Although our proposed method occasionally exhibits lower performance compared to the Weight Balancing (WB) technique, it typically shows improvements across most metrics. This is particularly evident in the ${F}_{1}$ score, which accounts for both precision and recall, where our method consistently demonstrates a noticeable improvement. For instance, in the context of the OPMD category's ${F}_{1}$ score, the WB technique achieved 0.7333, and the Oversampling technique reached 0.5600. Our proposed method, on the other hand, attained a score of 0.7692.

Our proposed mosaic-based soft labeling may demonstrate the ability to achieve similar performance to using a class-balanced dataset even when dealing with a class-imbalanced dataset. Jubair et al. [11] and Heo et al. [12] performed binary classification on imbalanced datasets with the existing methods, such as oversampling and weight-balancing. Jubair et al. used 716 images, which consisted of 236 cancerous images and 480 benign images. Heo et al. used 5,576 images, including 3,635 non-cancer images and 1,941 cancer images. Their models achieved an accuracy of 85.0% and 84.7%, respectively. Although the data is different and so not directly comparable, we tackled a more challenging three-class classification using an imbalanced dataset and achieved an accuracy of 94.44%. Notably, our method was able to achieve comparable performance on the imbalanced dataset as the previous study obtained on the balanced dataset with OPMD [15]. Sharma et al. [15] achieved an accuracy of 76% in three-class classification using a conventional CNN with a class-balanced dataset. Their study utilized 329 images, comprising 106 normal, 102 OPMD, and 121 cancer images. Although their dataset was balanced, the ${F}_{1}$ score of OPMD was 0.74. In contrast, in our study, we achieved an ${F}_{1}$ score of 0.7692 for OPMD, despite using a class-imbalanced dataset.

We have proposed a new technique that utilizes mosaic-based soft labeling which provides the combined benefits of soft labeling, oversampling, and traditional image manipulation-based augmentation. Two-stage CL was used to apply mosaic-based soft labeling into deep learning-based tongue cancer classification.

The soft labeling technique replaces the one-hot ground truth with smoothed labeling, known to enhance generalization performance. Szegedy et al. demonstrated a 0.2% reduction in error for the ImageNet Large Scale Visual Recognition Challenge (ILSVRC) 2012 by incorporating soft labeling into training [44]. In our proposed method, each component of the label represents the area occupied by each class's corresponding regions, intrinsically having the effect of soft labeling.

Oversampling and undersampling are the resampling techniques [45–47] that are commonly used to address imbalanced classifications [48, 49]. Oversampling increases the minority class instances, whereas undersampling reduces the majority class instances. Our proposed method has an intrinsic oversampling effect owing to random sampling with replacement and allocation of larger areas to the minority class during dataset generation. This allows users to adjust the oversampling ratio by setting the number of synthesized images and the area of each patch in the synthesized image.

In our experiments, the models trained only with mosaic-based soft labeling images performed worse, apparently because the cancer- or OPMD-independent patterns present in the synthesized images had a negative impact during training. Therefore, conventional image transformations, such as rotation and translation, are necessary to ensure that the network does not focus on useless patterns that are independent of the region of interest, such as a grid pattern, which is a cross-shaped line between images in a synthesized image.

The dataset employed in this study comprised only 1,810 images of tongues and was highly imbalanced. Specifically, the OPMD class accounts for only 7.8% of the data. Generally, OPMD patients are more prevalent than cancer patients, so cancer images are expected to be less abundant. However, in this study, we collected imaging data from patients who visited the outpatient clinic of a professor specializing in cancer surgery (JHL). Due to the higher frequency of requests for patients requiring cancer surgery, it seemed that there are fewer imaging instances for OPMD compared to cancer. This can result in a poor performance in practical clinical situations. Using the proposed method, along with additional OPMD data collection, we expect that there will be a performance improvement compared to existing data augmentation techniques. We also anticipate that further performance gains can be achieved using the proposed data augmentation method to generate additional images when combined with other techniques to address class imbalance. Based on our experimental results, in the case of training with class-imbalanced datasets, the oversampling technique tends to be more effective compared to the undersampling technique. Additionally, incorporating the weight-balancing technique may lead to some improvements in performance. For those seeking a more balanced and stable outcome, our mosaic-based soft labeling method could prove beneficial, though the degree of improvement might vary. Moreover, if additional data collection is undertaken for classes with fewer samples, better performance can be expected. As highlighted by Halevy et al. [50], collaborative efforts in collecting more clinical tongue images could offer a fundamental solution to the challenges of data scarcity and class imbalance. However, the exact impact of such efforts on performance would require further exploration.

Although this study presents a new method for enhancing tongue cancer classification and provides some practical to remedy class imbalanced problem, it has certain limitations. The first limitation of our study is that it relies solely on tongue images. Oral cancer may also appear in other regions. Additionally, our study did not account for lesion location in the training and analysis of the model. Second, the lower performance in OPMD may be influenced by the limited sample size, but it could also be attributed to the diverse subcategories within OPMD. Because it is not merely a matter of an imbalanced dataset, additional research is required to understand the effect of diverse subcategories within OPMD both on deep learning model training and performance. Thirdly, in this study, only images acquired by one professor's camera were utilized. However, for the detection of images in more universal scenarios, it may be necessary to have images from various environments. As OPMD patients receive care not only from professors in oral and maxillofacial surgery but also from professors in oral medicine, we plan to collaborate with these professors in the future to gather imaging data collaboratively. Fourthly, if a sufficiently large and representative dataset is available, there may not be a significant performance difference between the conventional approach and our proposed method in the ideal situation. Previous studies have reported that the choice of training technique or algorithm has minimal impact on performance when the dataset is representative [50, 51]. In contrast, when the dataset significantly lacks representativeness, training deep learning models effectively becomes a challenge, even when employing our mosaic-based soft labeling technique. This requires further study to identify the range of dataset representativeness where mosaic-based soft labeling exceeds the performance of traditional methods. An initial step in such research could involve quantifying the relationship between the size of the dataset and the effectiveness of mosaic-based soft labeling.

## Conclusion

In this study, we propose a novel data augmentation technique called ‘mosaic-based soft labeling’ to approach optimal performance on an imbalanced tongue cancer dataset. We introduced a CL strategy to generate synthetic mosaic images and improve the classification performance of tongue cancer. In the first stage, we trained a tongue cancer classification model using an imbalanced dataset. Utilizing information from the trained network, we synthesized and labeled mosaic images. The model in the second stage was then trained by incorporating synthetic mosaic images. The proposed approach improved the classification performance of tongue lesions compared to previous methods, such as oversampling and weight balancing. However, it fell somewhat short of our initial expectations. Nevertheless, it establishes a foundation for future research on effectively learning from imbalanced data, a common challenge in many diagnostic applications.

## Data Availability

Owing to privacy concerns, the datasets generated and/or analyzed during the current study are available from the corresponding author upon reasonable request.

## Abbreviations

OPMD: Oral potentially malignant disorders
RICAP: Random Image Cropping and Patching
CNN: Convolutional neural network
CL: Curriculum learning
TP: True Positive
TN: True Negative
FP: False Positive
FN: False Negative
NCL: Noncancerous lesions
ROI: Region Of Interest
